# Case Report: Intravascular lithotripsy combined with a new-generation 3-in-1 carotid stent for calcified carotid artery stenosis

**DOI:** 10.3389/fsurg.2026.1929139

**Published:** 2026-09-09

**Authors:** William J. P. Ertl, Uttam Verma, Hakeem J. Shakir, Shyian Jen

**Affiliations:** Department of Neurosurgery, University of Oklahoma Health Sciences Center, Oklahoma City, OK, United States

**Keywords:** calcified plaque, carotid artery stenting, carotid stenosis, case report, intravascular lithotripsy, ischemic stroke

## Abstract

**Background:**

Symptomatic carotid atherosclerotic stenosis is a major cause of ischemic stroke. In patients with heavily calcified plaque and limited physiologic reserve for open revascularization, conventional carotid artery stenting may fail because rigid calcium prevents adequate luminal expansion. Intravascular lithotripsy (IVL) is an adjunctive plaque-modification strategy for selected calcified carotid lesions, although evidence remains limited and carotid IVL is investigational.

**Case presentation:**

An 83-year-old man with hyperlipidemia, hypertension, hypothyroidism, protein S deficiency, prior myocardial infarction, and lower-extremity deep vein thrombosis on apixaban presented with acute ischemic stroke manifested by dysarthria, global aphasia, and right hemiparesis. Brain MRI showed acute left-hemisphere borderzone infarcts involving deep white matter and basal ganglia, consistent with ipsilateral carotid atheroembolism and/or relative hypoperfusion. CTA and cerebral angiography demonstrated approximately 60% left internal carotid artery stenosis, with heavy concentric calcification spanning a lesion length greater than 10 mm at the carotid bifurcation.

**Management and outcome:**

After multidisciplinary review of carotid endarterectomy, TCAR, and transfemoral options, IVL-assisted carotid angioplasty and stenting under continuous embolic protection was selected given advanced age, prior myocardial infarction, acute neurologic impairment, and limited functional reserve. Using distal embolic protection and proximal balloon-guide flow arrest, IVL was performed across the calcified plaque, followed by deployment of a new-generation integrated 3-in-1 carotid stent. Angiographic stenosis improved from approximately 60% to approximately 20% by NASCET methodology without periprocedural stroke, vasospasm, dissection, hemodynamic instability, or access-site complication. Admission NIHSS was 29; discharge NIHSS and mRS were 6 and 3, respectively. At approximately 1-month follow-up he was alert and oriented ×3 with fluent language and good comprehension. No further antithrombotic regimen switch occurred during outpatient follow-up, and no perioperative complications were reported.

**Conclusion:**

In this elderly patient with acute ischemic stroke and heavily calcified symptomatic carotid stenosis, IVL with angioplasty and a 3-in-1 carotid stent achieved early technical success without periprocedural complication and with short-term clinical stability. This case supports further investigation of IVL-based carotid revascularization in carefully selected patients who are poor candidates for open surgery and highlights the need for standardized follow-up, neurologic outcome reporting, and prospective evaluation of long-term patency and stroke prevention.

## Introduction

Stroke is well-characterized as one of the leading causes of death in developed countries and the leading cause of morbidity worldwide, with the majority being ischemic in nature. Carotid atherosclerotic disease is a major cause of ischemic stroke, primarily through artery-to-artery embolism and cerebral hypoperfusion from flow-limiting stenosis. These events reflect the clinical consequences of carotid atherosclerosis, which arises through multiple overlapping pathogenic pathways. Progressive endothelial injury and intimal lipid accumulation lead to gradual luminal narrowing that may reduce cerebral perfusion and contribute to transient ischemic attack or ischemic stroke.

Traditional nonsurgical approaches can be used for mild and moderate asymptomatic stenosis, however severe or symptomatic stenosis causing neurologic deficits often requires revascularization, typically by carotid endarterectomy (CEA), carotid artery stenting (CAS), or, more recently, transcarotid artery revascularization (TCAR). Although these procedures are effective, heavy calcification in advanced-age patients, those with prior neck surgery or radiation, or others who are poor candidates for open surgery may limit feasibility due to rigid calcium that can prevent adequate plaque removal or stent expansion (1–3). This technical gap has motivated interest in adjunctive plaque-modification strategies for selected calcified carotid lesions.

Intravascular lithotripsy (IVL) with balloon angioplasty and stenting has been reported as a potential approach to such plaques. Previously used for calcified coronary disease and for urinary stone fragmentation, IVL uses a specialized balloon catheter and acoustic pressure waves to fracture intimal and medial calcium while limiting injury to adjacent soft tissue, thereby facilitating subsequent angioplasty and stent expansion (4, 5).

Although prior reports support feasibility, published experience remains limited, and carotid IVL remains off-label. In addition, to our knowledge, IVL has not previously been reported together with a new-generation 3-in-1 carotid stent that integrates embolic protection, a post-dilation balloon, and a self-expanding stent. Here, we describe IVL-assisted angioplasty with a 3-in-1 carotid stent for heavily calcified symptomatic carotid stenosis in an elderly patient with acute ischemic stroke.

## Patient information and assessment

**Figure 1:**
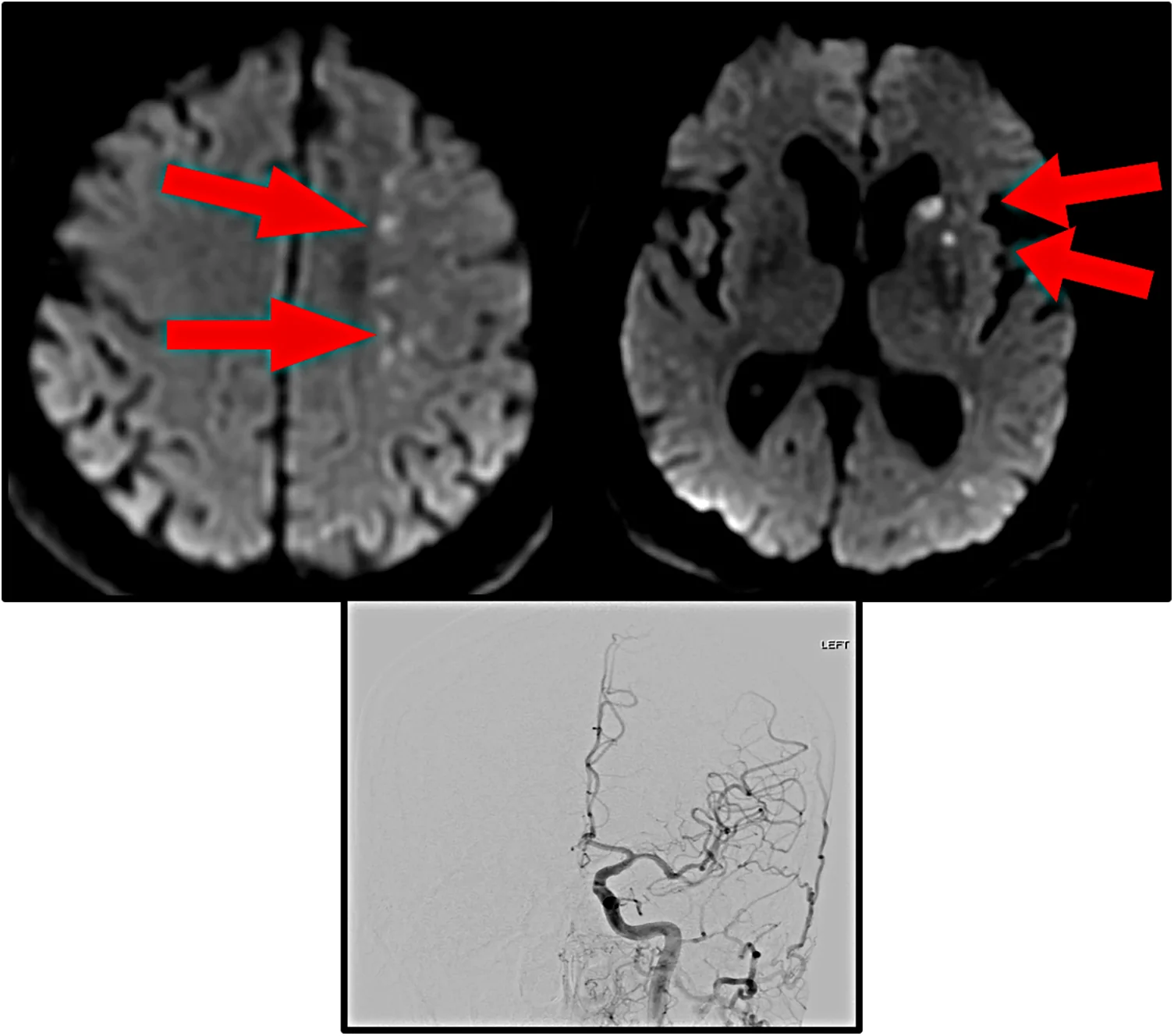
Top: MRI brain with diffusion restriction in the left hemisphere due to a heavily calcified atheroembolism from the left ICA stenosis. Bottom: Left AP Townes intracranial angiography with anterograde filling of the left MCA and left ACA branches.

This case report was prepared in accordance with the CARE guidelines (6). An 83-year-old white man with hyperlipidemia, hypertension, hypothyroidism, protein S deficiency, lower-extremity deep vein thrombosis (DVT), and prior myocardial infarction, managed with apixaban 2.5 mg twice daily, was admitted with acute ischemic stroke manifesting as dysarthria, global aphasia, and right-sided hemiparesis. The presumed pre-hospital modified Rankin Scale (mRS) score was 1, and the admission NIHSS was 29. MRI with diffusion restriction demonstrated multiple small acute left-hemisphere borderzone infarcts involving deep white matter and basal ganglia, consistent with ipsilateral carotid atheroembolism and/or relative hypoperfusion, without large-vessel intracranial occlusion (Figure 1). Our patient was not a candidate for intravenous thrombolysis because of recent DOAC use or for mechanical thrombectomy because no large-vessel occlusion was present. The patient was loaded with aspirin and ticagrelor and subsequently taken to the angiographic suite.

**Figure 2:**
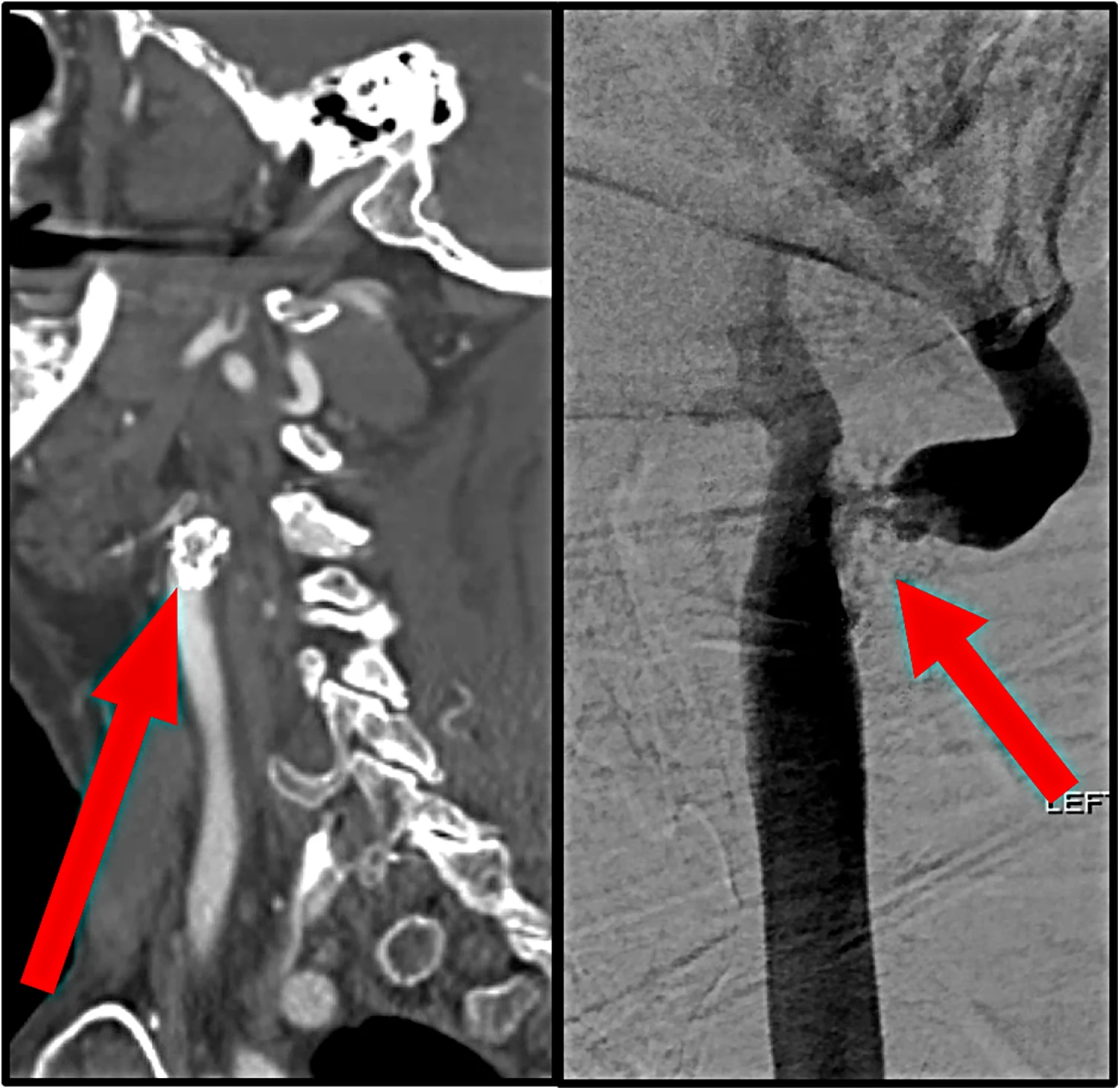
Pre-revascularization characterization of the left carotid bifurcation lesion. Left: Sagittal CTA of the neck demonstrating heavy near-circumferential calcification of the distal left common carotid army)/ and proximal internal carotid artery (arrow). Right: Selective left common carotid digital subtraction angiography confirming approximately 60% stenosis by visual NASCET estimate at the calcified carotid bifurcation lesion (arrow).

CTA of the neck and diagnostic cerebral angiography demonstrated approximately 60% stenosis of the proximal left ICA by NASCET methodology, with heavy, deep, near-circumferential calcification spanning a lesion length greater than 10 mm at the carotid bulb with a minimal luminal diameter (MLD) of 2.0 mm (Figure 2). Reference vessel diameters measured approximately 6.7 mm in the CCA, 5.1 mm in the ICA, and 4.0 mm in the ECA. There was no angiographic evidence of ulceration, intraluminal thrombus, tandem cervical stenosis, or intracranial atherosclerosis. Contralateral carotid disease was absent. Intracranial vessel runs were otherwise normal. Diagnostic reasoning favored the left carotid bifurcation lesion as the ipsilateral culprit based on acute left-hemisphere borderzone and basal ganglia infarcts, consistent with ipsilateral carotid atheroembolism and/or relative hypoperfusion, ipsilateral calcified carotid plaque, and absence of intracranial large-vessel occlusion. Electrocardiography, inpatient telemetry, and transthoracic echocardiography with bubble study were obtained during the index admission and were normal, reducing concern for a competing cardioembolic source. No major challenges were encountered during initial diagnostic evaluation with MRI, CTA, and cerebral angiography.

A multidisciplinary discussion among neurosurgery, vascular surgery, neurology, and interventional neuroradiology was held to review the risks and benefits of CEA, TCAR, and transfemoral CAS with adjunctive IVL. Although absolute stenosis was moderate rather than high-grade, revascularization was favored because of a clearly symptomatic ipsilateral lesion with borderzone/basal ganglia infarct pattern, heavy concentric calcification raising concern for ongoing atheroembolism and limited response to conventional angioplasty alone, and high risk of early recurrent ipsilateral ischemia if the culprit lesion were left untreated. Medical management alone and delayed revascularization were considered but judged less appropriate given recent infarction attributable to calcified carotid disease. CEA was considered high risk given advanced age, prior myocardial infarction, acute neurologic impairment, and limited physiologic/functional reserve for open surgery under general anesthesia. TCAR was discussed as an alternative for high-surgical-risk patients that can provide flow reversal; however, transfemoral IVL-assisted CAS was selected to avoid a cervical cutdown in a patient on apixaban plus loaded dual antiplatelet therapy and to permit local rather than general anesthesia in a frail, elderly, post-MI patient. While TCAR can be performed under local anesthesia, awake neurologic monitoring would have been limited by global aphasia at the time of consent. IVL was chosen specifically to fracture the heavy concentric calcium and allow adequate stent expansion. Informed consent for off-label IVL-assisted transfemoral CAS was obtained after shared decision-making with the patient's spouse as a communication facilitator due to global aphasia.

## Timeline

The chronology of diagnostic evaluation, multidisciplinary decision-making, IVL-assisted carotid revascularization, and follow-up is summarized in Table 1 below.

**Table 1:** Timeline of the episode of care from index hospitalization for acute ischemic stroke through approximately 90-day follow-up after IVL-assisted carotid artery stenting.

Time	Focus	Key findings/intervention
Day 0	Diagnostics/follow-up	Index presentation 83-year-old man with hyperlipidemia, hypertension, hypothyroidism, protein S deficiency, prior MI, and lower-extremity DVT on apixaban Acute ischemic stroke: dysarthria, global aphasia, and right hemiparesis NIHSS 29, mRS 1 (presumed)
Day 0	Diagnostics/follow-up	Diagnostic brain MRI Multiple small acute left-hemisphere borderzone infarcts involving deep white matter and basal ganglia, consistent with ipsilateral carotid atheroembolism and/or relative hypoperfusion (Figure 1) No large-vessel intracranial occlusion
Day 0	Diagnostics/follow-up	CTA/cerebral angiography ∼60% left ICA stenosis (NASCET methodology) Heavy near-circumferential calcification; lesion length >10 mm w/ MLD approx. 2.0 mm (Figure 2) Reference diameters: CCA 6.7 mm, ICA 5.1 mm, ECA 4.0 mm No ulceration, thrombus, tandem, contralateral, or intracranial atherosclerosis Favored as ipsilateral culprit; EKG, telemetry, and TTE with bubble study normal
Day 1–2	Decision/intervention	Multidisciplinary review & consent MDT reviewed CEA vs TCAR vs TF IVL-CAS TF IVL-CAS selected over CEA (perioperative risk) TCAR deferred (cervical cutdown on anticoagulation/DAPT; local anesthesia; aphasia limiting awake monitoring) Off-label consent with spouse facilitator
Day 2	Decision/intervention	IVL-assisted angioplasty & stenting • Local anesthesia; TF access; 8 Fr Walrus BGC + Emboshield distal EPD • No predilation • Shockwave C2+ IVL 4.0 × 12 mm at 3 atm (5 × 10 s), sized to ICA reference diameter; residual ∼37% • Neuroguard IEP 9/7/8-mm; integrated post-dilation to 5 atm • Final stenosis ∼20% by NASCET criteria (Figure 3) • Procedure duration 1 h 51 min; fluoroscopy 94.3 min; contrast ∼100 mL • No periprocedural stroke, dissection, or hemodynamic/access complication
Postprocedure hospital course	Diagnostics/follow-up	Inpatient course after revascularization Immediate post-procedure: aspirin held; ticagrelor 90 mg twice daily + apixaban 2.5 mg twice daily briefly Index admission: revised to DAPT (aspirin 81 mg daily + ticagrelor 90 mg twice daily); apixaban held for early bleeding-risk mitigation Discharged on DAPT; apixaban remained held through reported follow-up; longer-term anticoagulation deferred to outpatient follow-up Hemorrhagic-transformation risk considered; platelet function testing not performed
∼1 month	Diagnostics/follow-up	Neurointerventional clinic follow-up (telehealth) Alert and oriented ×3; fluent language with good comprehension; ocular movements intact; face symmetric; hearing intact No TIA, stroke, readmission, or death Ticagrelor-associated dyspnea noted; improved after dose adjustment Formal NIHSS/mRS not consistently documented at this telehealth visit
∼90 days	Diagnostics/follow-up	∼90-day clinical & duplex follow-up Global aphasia resolved; continued clinical improvement No TIA, stroke, readmission, or death Patent stent without significant in-stent restenosis (post-CAS criteria; Supplementary Figure S1) ICA/CCA PSV ratio 2.0; EDV ratio 1.5 No postoperative DWI; silent embolization could not be assessed

CAS, carotid artery stenting; CCA, common carotid artery; CEA, carotid endarterectomy; DAPT, dual antiplatelet therapy; DVT, deep vein thrombosis; EDV, end-diastolic velocity; EPD, embolic protection device; ICA, internal carotid artery; IVL, intravascular lithotripsy; MI, myocardial infarction; MLD, minimal luminal diameter; mRS, modified Rankin Scale; NIHSS, National Institutes of Health Stroke Scale; PSV, peak systolic velocity; TCAR, transcarotid artery revascularization; TF, transfemoral; TTE, transthoracic echocardiography.

## Therapeutic intervention

After local anesthetization, an 8 Fr Pinnacle sheath (Terumo Medical, USA) was placed over a 0.035″ Amplatz stiff wire (Boston Scientific, USA) in the right common femoral artery. An 8 Fr Walrus balloon guide catheter (Q'Apel Medical, USA) was advanced into the distal left common carotid artery. Digital subtraction angiograms of the left anterior cervical circulation confirmed approximately 60% stenosis of the proximal ICA secondary to concentrically calcified plaque greater than 10 mm in length at the carotid bulb (Figure 2). Intracranial vessel runs were otherwise normal (Figure 1).

The stenotic segment was crossed with a 0.014″ BareWire Workhorse Filter Delivery Wire platform under roadmap assistance and maintained throughout IVL and stenting. An Emboshield embolic protection device (Abbott Cardiovascular, USA) was advanced over this wire into the distal left ICA and deployed. After placement confirmation, the balloon guide catheter was inflated in the distal CCA for proximal flow arrest. No hemodynamic intolerance was observed during balloon occlusion; neurologic status specifically during flow arrest was not separately documented. No conventional predilation was performed prior to IVL.

**Figure 3:**
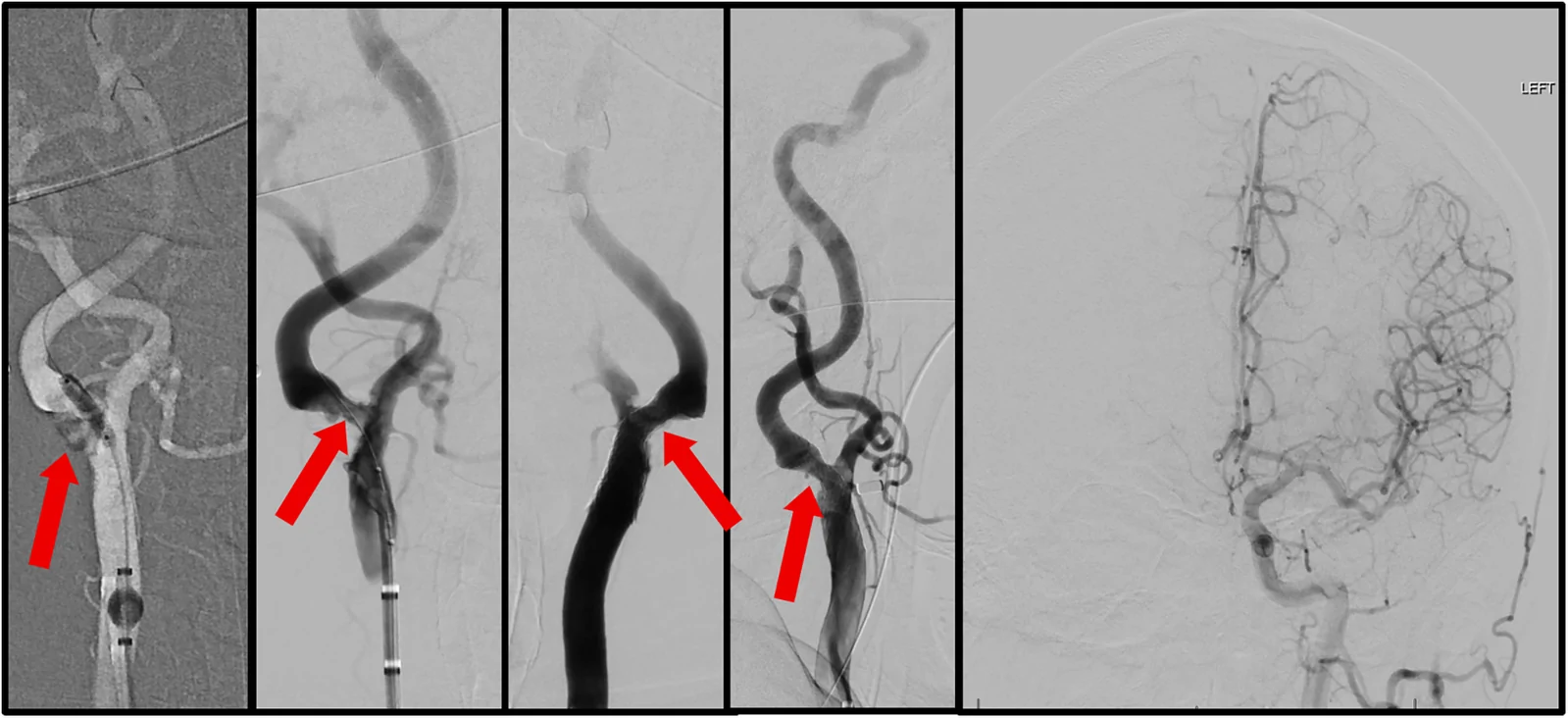
Procedural digital subtraction angiography of IVL-assisted left carotid stenting (left to right). (1) Pre-IVL working projection with a 4.0 × 12 mm Shockwave C2+ balloon across the calcified left carotid bifurcation plaque under distal embolic protection (arrow at lesion), immediately before proximal balloon-guide flow arrest. (2) Immediate post-IVL angiogram after 5 cycles at 3 atm showing improved luminal caliber with residual stenosis of approximately 37% (arrow). (3) Selective post-stent left anteroposterior and (4) lateral common carotid angiography after Neuroguard IEP 9/7/8-mm deployment and integrated balloon post-dilation to 5 atm, demonstrating approximately 20% residual stenosis by visual NASCET estimate (arrows at residual stenosis) with good wall.

An IVL 4.0 × 12 mm C2+ balloon catheter (Shockwave Medical, USA), selected based on the approximately 5.1 mm ICA reference diameter, was positioned across the calcified plaque, inflated to 3 atm, and treated with 5 cycles of 10 s each (Figure 3). Continuous arterial pressure monitoring and cardiac telemetry were maintained throughout all five IVL cycles, and atropine was available per protocol. After deflation of the balloon catheter and follow-up angiography, the balloon catheter was removed. A residual stenosis of approximately 37% was observed post-IVL (Figure 3).

A 9/7/8-mm tapered Neuroguard IEP 3-in-1 stent system (Contego Medical, USA; distributed by Medtronic) was then advanced over the 0.014″ distal protection device wire and positioned across the stenosis. The self-expanding stent was deployed, and post-stenting angioplasty was performed with the integrated balloon catheter inflated to 5 atm. The stent system was then removed, the embolic protection device was recaptured, and final digital subtraction angiography demonstrated improvement from approximately 60% to approximately 20% residual stenosis (Figure 3). Total procedural duration was 1 h 51 min; fluoroscopy time was 94.3 min, and iodinated contrast volume was approximately 100 mL as recorded in the procedural log. No deviations to the planned procedure were reported.

After the procedure, the patient was monitored in the hospital. To avoid early triple therapy after recent infarction, aspirin was not continued in the immediate post-procedure period; ticagrelor 90 mg twice daily plus apixaban 2.5 mg twice daily was used briefly for stent protection and prior lower-extremity DVT in the setting of protein S deficiency. This regimen was then revised during the index admission to dual antiplatelet therapy with aspirin 81 mg daily and ticagrelor 90 mg twice daily, and apixaban was held to reduce early post-stent bleeding risk. The patient was discharged to inpatient rehabilitation on this DAPT regimen. Apixaban remained held through the reported outpatient follow-up interval, with no further antithrombotic regimen switch; longer-term anticoagulation for protein S deficiency and prior DVT was deferred to individualized outpatient clinical follow-up after the early post-stent period. NIHSS/mRS scores on discharge were 6 and 3, respectively. Technical success was defined *a priori*, consistent with prior carotid IVL/CAS reporting standards, as delivery of IVL across the target lesion and stent deployment with ≤30% residual stenosis (7). No evidence of vasospasm, vessel dissection, or distal emboli was observed. No bradycardia, hypotension, or arrhythmia occurred. Temporary transvenous pacing was not initiated because the patient remained hemodynamically stable without symptomatic or sustained bradycardia.

## Follow-up

The patient remained free of periprocedural stroke, TIA, or access-site complication. At approximately 1-month neurointerventional clinic follow-up via telehealth, he was alert and oriented ×3, with fluent language and good comprehension, intact ocular movements, symmetric facial expression, and intact hearing. No interval TIA, stroke, readmission, or death had occurred. Ticagrelor-associated dyspnea was noted and later improved after dose adjustment. Residual dysesthesia attributed to the index stroke was treated with amitriptyline. Formal NIHSS and mRS were not consistently documented at every outpatient visit because of travel distance and limited clinic-based assessment due to telehealth communication.

At approximately 90-day follow-up, global aphasia had resolved, and there had been no TIA, stroke, readmission, or death. Duplex ultrasound showed a patent stent without hemodynamically significant in-stent restenosis using validated post-CAS in-stent velocity criteria (Supplementary Figure S1) (8). Peak systolic velocity/end-diastolic velocity values (cm/s) were: proximal CCA 74/9, distal CCA 68/13 (stented), proximal ICA 116/13 (stented), mid ICA 137/20, and distal ICA 74/17. ICA/CCA PSV and EDV ratios were 2.0 and 1.5, respectively. Postoperative DWI was not obtained because the patient remained neurologically stable with visible clinical improvement and no new focal deficits; silent embolization therefore could not be assessed.

## Discussion

Previous case reports and series have described IVL with angioplasty and stenting in conjunction with distal embolic protection. To our knowledge, this report represents the first use of IVL with the Neuroguard IEP 3-in-1 system and adjunctive distal EPD during transfemoral CAS in the acute ischemic stroke setting. Heavily calcified carotid plaque can limit luminal gain with conventional angioplasty and is associated with residual stenosis and adverse periprocedural risk after stenting (9). High-pressure balloon angioplasty and atherectomy are alternative plaque-modification strategies, but atherectomy may carry perforation risk, whereas IVL is designed to fracture intimal and medial calcium while limiting soft-tissue injury (10). In our patient, IVL without predilation facilitated subsequent stent expansion to approximately 20% residual stenosis.

Although CEA remains an established option for many calcified carotid lesions and TCAR can provide flow reversal, the perioperative risk profile in this frail, elderly, post-MI patient with acute neurologic deficits favored a transfemoral endovascular approach under local anesthesia. Compared with atherectomy, IVL is intended to disrupt deep calcium while limiting soft-tissue injury and perforation risk (10). Selection among CEA, TCAR, and transfemoral CAS remains individualized. Transfemoral CAS with continuous distal filter protection plus proximal balloon-guide flow arrest was chosen to permit IVL-based plaque modification in the neuroangiography suite under local anesthesia. An additional practical advantage of a 3-in-1 stent is integration of embolic protection, a post-dilation balloon, and a self-expanding stent into one device, reducing device exchanges during the procedure.

The Emboshield filter (approximately 30 µm pores) was maintained across IVL and stent deployment to capture debris during calcific microfracture, in addition to the Neuroguard system's integrated protection (approximately 40 µm), reflecting a deliberate dual-protection strategy rather than reliance on either device alone. Distal embolic protection is commonly used to reduce periprocedural embolic risk (11, 12). Prior carotid IVL series have generally reported low rates of major periprocedural stroke, with transient ischemic attack among the more frequently described events (2).

To contextualize this case, we reviewed published English-language reports of carotid IVL used as an adjunct to CAS or TCAR. A PubMed/MEDLINE search using the terms “intravascular lithotripsy,” “shockwave,” and “carotid” was last performed on 21 July 2026. Prior series and case reports have described IVL predominantly in severely calcified and often high-grade lesions (≥70%), across both transfemoral and TCAR approaches, with generally low reported rates of major periprocedural stroke but heterogeneous embolic-protection strategies and follow-up intervals. To our knowledge, after this search, the present report is the first description of IVL combined with the Neuroguard IEP 3-in-1 system and adjunctive distal filter protection during transfemoral CAS in the acute ischemic stroke setting. Supplementary Table S1 summarizes selected published carotid IVL experiences and highlights differences from the present case, including acute stroke presentation, moderate (approximately 60%) stenosis, dual proximal/distal protection, an integrated 3-in-1 stent platform, and approximately 90-day duplex follow-up (13).

These findings are preliminary and should be interpreted cautiously. A single case with incomplete formal neurologic scores at every time point, no post-procedure DWI to exclude silent embolization, short imaging follow-up, and off-label carotid IVL cannot demonstrate superiority to CEA, TCAR, or conventional CAS. Residual stenosis was estimated visually using NASCET methodology rather than quantitative angiography. Objective frailty metrics were not recorded. Long-term restenosis and stroke-prevention efficacy cannot be inferred from 90-day follow-up alone. Although clinical follow-up occurred at approximately 1 month, formal NIHSS/mRS were not consistently documented at that visit. Postoperative DWI was not obtained at approximately 90 days because of clinical improvement without new neurologic deficits; this remains a limitation. This report describes routine clinical care rather than a prospective research protocol. Future studies with larger cohorts and a broader range of stenosis severity are needed to better characterize perioperative risk, clarify risk–benefit, and evaluate restenosis rates.

## Conclusion

In this 83-year-old patient with acute ischemic stroke and heavily calcified symptomatic left carotid bifurcation stenosis, IVL-assisted angioplasty and Neuroguard IEP stent deployment achieved successful luminal reconstruction without periprocedural complication and demonstrated stent patency at 90 days. These findings suggest that IVL-assisted carotid revascularization may be a technically feasible endovascular option in carefully selected patients who are poor candidates for open endarterectomy, when continuous embolic protection and multidisciplinary stroke evaluation are used. In a single case, technical success is descriptive and does not imply comparative efficacy or safety. Broader conclusions regarding neurologic recovery, durability, and superiority to standard revascularization strategies cannot be drawn. Prospective multicenter evaluation is needed.

## Patient perspective

According to the patient's spouse, who facilitated communication because of aphasia during the acute hospitalization, the family understood that open surgery carried higher perioperative risk given his age and cardiac history, preferred a less invasive option, and observed speech improvement through early recovery and outpatient therapy. They were thankful for the expertise provided by the neurosurgery team and the thoroughness they took into planning his operation and post-operative care.

## Data Availability

The original contributions presented in the study are included in the article/Supplementary Material, further inquiries can be directed to the corresponding author/s.
