# Supplementary material for: Case Report: Intravascular lithotripsy combined with a new-generation 3-in-1 carotid stent for calcified carotid artery stenosis
Source: Front Surg. 2026 Sep 9;13:1929139. doi: 10.3389/fsurg.2026.1929139 (PMC13597735; doi:10.3389/fsurg.2026.1929139)
Supplement: Supplementary file 1 [file Datasheet1.docx]

Supplementary Figure 1. Approximately 90-day duplex ultrasound follow-up demonstrating a patent carotid stent without hemodynamically significant in-stent restenosis by validated post-CAS velocity criteria (segmental color/spectral Doppler panels).

**Supplementary Table 1.** *Selected published reports of carotid IVL as an adjunct to CAS or TCAR*

| **Author / year** | **n** | **Approach** | **Setting** | **Stenosis / calcium** | **Protection** | **Stent platform** | **Follow-up** | **Key difference vs present case** |
| --- | --- | --- | --- | --- | --- | --- | --- | --- |
| Vadalà et al., 2020 | 3–8* | TF-CAS | Elective / high-risk | Severely calcified; high-grade | Distal EPD (variable) | Conventional CAS stents | Short-term | Early experience; not acute stroke; not Neuroguard |
| Giannopoulos et al., 2021 | 21 | TF-CAS (multicenter) | High surgical risk; mixed cerebrovascular history | Mean ~82% stenosis; heavily calcified | EPD in all cases | Conventional CAS stents | Median 6 mo (1–12) | Higher-grade stenoses; not acute-stroke index; not Neuroguard |
| Dadashzadeh et al., 2023 | Case report | TCAR | High-risk calcific ICA | Severe calcific ICA disease | TCAR flow reversal | TCAR stent platform | Short-term | TCAR rather than TF-CAS; not Neuroguard |
| DiLosa et al., 2024 | 59 | TCAR (multicenter) | High-risk patients | Severe calcification limiting TCAR | TCAR flow reversal | TCAR platforms | 30-day neurologic outcomes emphasized | TCAR cohort; not Neuroguard; not acute-stroke TF-CAS |
| Abreu et al., 2025† | 15 pts / 17 proc. | TF-CAS | Symptomatic or asymptomatic HCCS | Median stenosis 73% (IQR 60–80%) | Dual: BGC + distal EPS | Conventional CAS stents | ISR reported to ~5 mo in 1 patient | Similar dual protection; higher median stenosis; not Neuroguard |
| Almadani et al., 2025 | 4 | TCAR | High-risk; coral-reef calcification | Severely calcified ICA/CCA | TCAR flow reversal | TCAR stent | Median 8 mo (6–12); patent stents | TCAR; severe/coral-reef lesions; not TF acute-stroke case |
| Present case | 1 | TF-CAS + IVL | Acute ischemic stroke (index admission, day 2) | ~60% concentric calcified stenosis (NASCET); lesion >10 mm | Distal Emboshield + proximal Walrus BGC (+ Neuroguard IEP) | Neuroguard IEP 3-in-1 | ~90-day duplex (Figure 4); patent stent / no significant ISR | — |
